# Characterization and Prognosis of Biological Microenvironment in Lung Adenocarcinoma through a Disulfidptosis-Related lncRNAs Signature

**DOI:** 10.1155/2023/6670514

**Published:** 2023-08-04

**Authors:** Zhuo Yang, Shenglan Cao, Fangli Wang, Kangming Du, Fang Hu

**Affiliations:** ^1^School of Nursing, Chengdu University of Traditional Chinese Medicine, Chengdu, Sichuan, China; ^2^Department of Cardiology, Hospital of Chengdu University of Traditional Chinese Medicine, Chengdu, Sichuan, China; ^3^Department of Cardiothoracic Surgery, Hospital of Chengdu University of Traditional Chinese Medicine, Chengdu, Sichuan, China; ^4^Obstetric Department, Hospital of Chengdu University of Traditional Chinese Medicine, Chengdu, Sichuan, China

## Abstract

**Background:**

The role of disulfidptosis-related lncRNAs remains unclear in lung adenocarcinoma.

**Methods:**

Analysis in R software was conducted using different R packages, which are based on the public data from The Cancer Genome Atlas (TCGA) database. The transwell assay was used to evaluate the invasion and migration abilities of lung cancer cells.

**Results:**

In our study, we identified 1401 lncRNAs significantly correlated with disulfidptosis-related genes (|Cor| > 0.3 and *P* < 0.05). Then, we constructed a prognosis model consisting of 11 disulfidptosis-related lncRNAs, including AL133445.2, AL442125.1, AC091132.2, AC090948.1, AC020765.2, CASC8, AL606834.1, LINC00707, OGFRP1, U91328.1, and GASAL1. This prognosis model has satisfactory prediction performance. Also, the risk score and clinical information were combined to develop a nomogram. Analyses of biological enrichment and immune-related data were used to identify underlying differences between patients at high-risk and low-risk groups. Moreover, we noticed that the immunotherapy nonresponders have higher risk scores. Meanwhile, patients at a high risk responded more strongly to docetaxel, paclitaxel, and vinblastine. Furthermore, further analysis of the model lncRNA OGFRP1 was conducted, including clinical, immune infiltration, biological enrichment analysis, and a transwell assay. We discovered that by inhibiting OGFRP1, the invasion and migration abilities of lung cancer cells could be remarkably hindered.

**Conclusion:**

The results of our study can provide directions for future research in the relevant areas. Moreover, the prognosis signature we identified has the potential for clinical application.

## 1. Introduction

Worldwide, lung cancer is one of the most common cancers, and its incidence is still increasing [1]. Known as a multifactorial disease, lung cancer involves both environmental and genetic factors [2]. Among all subtypes, lung adenocarcinoma (LUAD) is the most predominant type. Despite significant medical advances, the prognosis of some patients with LUAD remains unsatisfactory [3]. Moreover, the pathogenesis of LUAD is largely unknown, and early diagnosis is still insufficient, which to some extent leads to treatment challenges for LUAD [4]. Consequently, identifying the genes linked to LUAD may improve the prognosis, diagnosis, and treatment of the disease.

Noncoding RNA with a length of over 200 bases is known as long noncoding RNA (lncRNA), which is famous for its widespread regulatory effects [5]. LncRNAs have multiple effect patterns including competitive endogenous RNA (ceRNA) mechanisms, protein-binding, transcriptional regulation, and so on [6]. In addition, many studies have indicated that lncRNAs may contribute to cancer development. For instance, Kong et al. discovered that lncRNA CDC6 could promote breast cancer progression through the ceRNA mechanism (miR-215/CDC6) [7]. Yuan et al. found that the lncRNA TLNC1 could accelerate liver cancer progression by hampering the p53 signaling pathway [8]. In lung cancer, Pan et al. found that the lncRNA JPX can promote lung cancer development through the miR-33a-5p/Twist1 axis [9]. Gao et al. noticed that lncRNA PCAT1 could inhibit radioimmune responses by regulating cGAS/STING signaling [10]. Hua et al. discovered that the lncRNA LINC01123 promotes proliferation and aerobic glycolysis by ceRNA mechanisms (miR-199a-5p/c-Myc axis) [11]. Recently, Liu et al. noticed a novel cell death form named “disulfidptosis” cell death, which is due to the aberrant accumulation of intracellular disulfides dependent on SLC7A11 [12]. Disulfidptosis is different from apoptosis and ferroptosis, which were previously uncharacterized. Therefore, prospective exploration of the lncRNA that regulates disulfidptosis can provide direction for future research in this field and reveal possible targets.

In our study, we identified 1401 lncRNAs significantly correlated with disulfidptosis-related genes (|Cor| > 0.3 and *P* < 0.05). Then, we constructed a prognosis model consisting of 11 disulfidptosis-related lncRNAs, including AL133445.2, AL442125.1, AC091132.2, AC090948.1, AC020765.2, CASC8, AL606834.1, LINC00707, OGFRP1, U91328.1, and GASAL1. Also, the risk score and clinical information were combined to develop a nomogram. Analyses of biological enrichment and immune-related data were used to identify underlying differences between patients in high-risk and low-risk groups. Moreover, we noticed that the immunotherapy nonresponders have higher risk scores. Meanwhile, patients at high risk responded more strongly to docetaxel, paclitaxel, and vinblastine. Furthermore, further analysis of the model lncRNA OGFRP1 was conducted, including clinical, immune infiltration, biological enrichment analysis, and a transwell assay. We discovered that by inhibiting OGFRP1, the invasion and migration abilities of lung cancer cells could be remarkably hindered.

## 2. Methods

### 2.1. Data Collection

The public data of LUAD patients were downloaded from the Cancer Genome Atlas database (TCGA)-KIRC project. The original transcriptome data form is “STAR-Counts.” The original clinical data form is “bcr-xml.” For the transcriptome data, the R code of the authors was used for data normalization. Clinical data were arranged using the Perl code. The distinction between coding genes and lncRNA is based on a reference genome file (GRCh38.gtf). Tumor stemness data were obtained from the previous study [13].

### 2.2. Collection of the Disulfidptosis-Related Genes and lncRNAs

The list of disulfidptosis-related genes was collected from the previous study conducted by Liu and their colleagues [12]. Correlation analysis was used to identify the disulfidptosis-related lncRNAs. For specific disulfidptosis-related genes, the lncRNAs with |cor| > 0.3 and *P*  <  0.05 were regarded as disulfidptosis-related lncRNAs. Cytoscape software was used to visualize the coexpression network of disulfidptosis-related genes-lncRNAs [14].

### 2.3. Construction of the Prognosis Model

As a first step, the patients were randomly assigned to training and validation cohorts. Genes associated with prognosis were identified using univariate Cox regression analysis (*P*  <  0.05). The final variables were optimized through the use of LASSO regression. Finally, multivariate Cox regression analyses were used to construct a prognosis model with the formula of “risk score = lncRNA A *∗* Coef A + lncRNA B *∗* Coef B + … + lncRNA N *∗* Coef N.”

### 2.4. Nomogram Plot

The nomogram was created by combining the risk score and clinical information to enhance its clinical applicability. A calibration plot was used to evaluate whether the nomogram predicted survival accurately.

### 2.5. Biological Enrichment Analysis

Gene set enrichment analysis (GSEA) was utilized to perform biological enrichment analysis based on multiple gene sets [15].

### 2.6. Immune-Related and Drug-Sensitivity Analysis

Multiple algorithms were used to quantify the immune infiltration status of the LUAD tissue microenvironment, including XCELL, CIBERSORT, EPIC, MCPCOUNTER, QUANTISEQ, and TIMER [16–20]. The single-sample GSEA (ssGSEA) was used to quantify the immune functions [21]. Using the tumor immune dysfunction and exclusion (TIDE) algorithm, the immunotherapy response was examined [22]. Data from the Genomics of Drug Sensitivity in Cancer (GDSC) database were used to analyze drug sensitivity [23].

### 2.7. Cell Culture and Quantitative Real-Time PCR (qPCR)

The lung cancer cell lines A549 and PC-9 used in this study were stored in our laboratory and cultured under conventional conditions (5% CO_2_ and 37°C). To produce cDNA, total RNA was extracted and reverse transcribed using a Universal RNA Extraction Kit (TaKaRa, Shanghai, China). The primers used for qPCR are shown in Supplementary file 1.

### 2.8. Cell Transfection

Cell transfection was performed using lipofectmine 2000 according to standard procedures. The shRNAs of OGFRP1 were designed and purchased from Guangzhou RiboBio (Guangzhou, China), and the target sequences were as follows: shRNA1, 5′-GGTGTTCACATGGCAGTAA-3′; shRNA2, 5′-GGATACTGAGAGTGCACAA-3′; and shRNA3, 5′-GCATTGACATGTTTGGCAT-3′.

### 2.9. Transwell Assay

According to standard procedures, transwell assays were performed on A549 and PC-9 cell lines [24].

### 2.10. Statistical Analysis

All statistical analyses were performed using R version 4.0.4 and GraphPad Prism 8 software. The threshold of statistical value is 0.05. Different statistical analysis methods were applied according to the data distribution form.

## 3. Results

**Figure 1:**
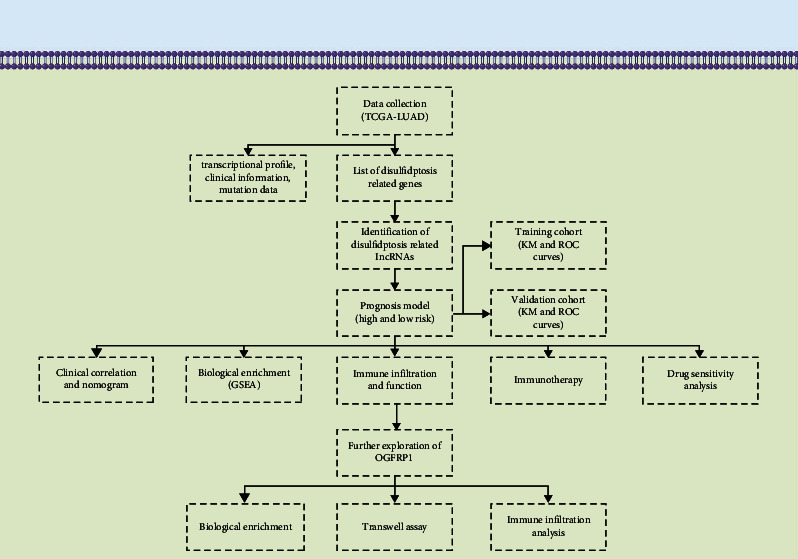
The flowchart of the whole study.

The flowchart of our study is shown in Figure 1.

### 3.1. Identification of Disulfidptosis-Related lncRNAs in LUAD

**Figure 2:**
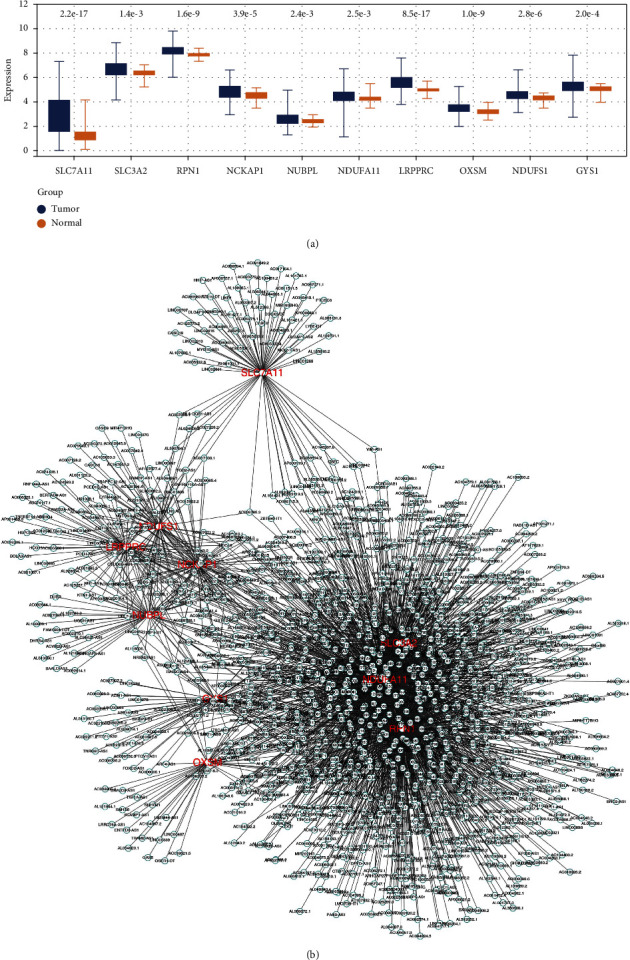
Identification of disulfidptosis-related genes and lncRNAs. (a) The disulfidptosis-related molecules from a previous study; (b) correlation analysis identified 1401 lncRNAs significantly correlated with disulfidptosis-related genes as disulfidptosis-related lncRNAs.

Based on the previous abovementioned studies, the genes SLC7A11, SLC3A2, RPN1, NCKAP1, NUBPL, NDUFA11, LRPPRC, OXSM, NDUFS1, and GYS1 were identified as the disulfidptosis-related genes. We found that all these disulfidptosis-related genes were upregulated in the tumor tissue, indicating their underlying effect on cancers (Figure 2(a)). A correlation analysis identified 1401 lncRNAs significantly correlated with disulfidptosis-related genes as disulfidptosis-related lncRNAs (Figure 2(b)).

### 3.2. Prognosis Model

**Figure 3:**
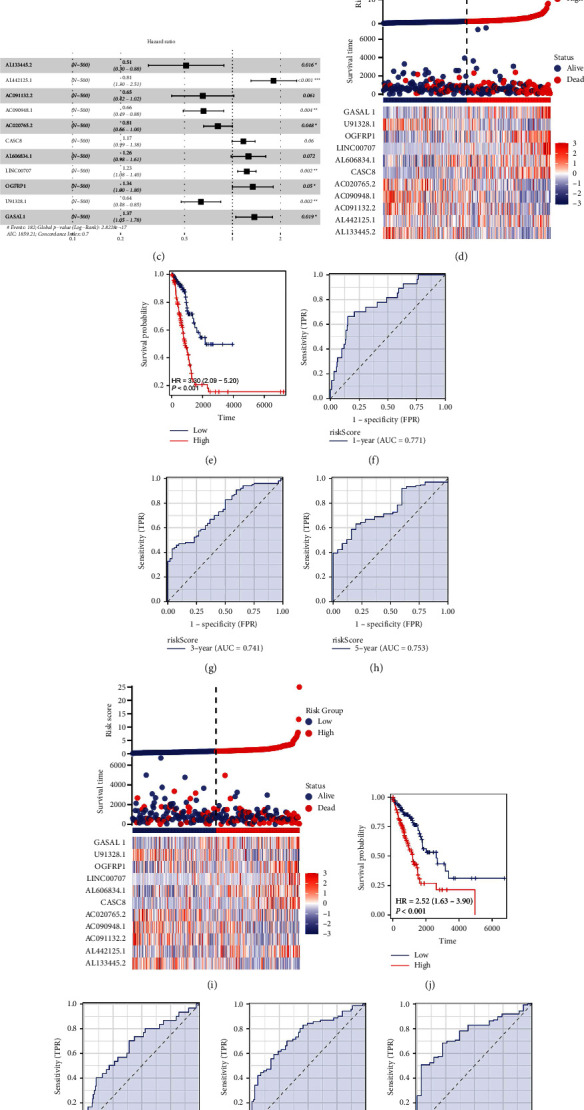
Construction of a prognosis model. (a, b) LASSO regression analysis; (c) multivariate cox regression analysis; (d) overview of our model in the training cohort; (e) KM survival curves of OS between high- and low-risk groups (training cohort); (f–h): ROC curves of our model in 1-, 3- , and 5-year survival (training cohort); (i) overview of our model in the validation cohort; (j) KM survival curves of OS between high- and low-risk groups (validation cohort); (k–m) ROC curves of our model in 1-, 3- , and 5-year survival (validation cohort).

Our first step was to divide the LUAD patients into 1 : 1 training and validation cohorts based on the TCGA data. First, we identified prognosis-related lncRNAs using univariate Cox regression analysis in the training cohort. Then, the LASSO regression analysis was applied to reduce data dimensions (Figures 3(a) and 3(b)). Ultimately, 11 disulfidptosis-related lncRNAs were identified for a prognosis model, including AL133445.2, AL442125.1, AC091132.2, AC090948.1, AC020765.2, CASC8, AL606834.1, LINC00707, OGFRP1, U91328.1, and GASAL1 (Figure 3(c)). The risk score of each patient was calculated with the formula of “risk score = AL133445.2 *∗* −0.6694 + AL442125.1 *∗* 0.5915 + AC091132.2 *∗* −0.4289 + AC090948.1 *∗* −0.4188 + AC020765.2 *∗* −0.2100 + CASC8 *∗* 0.1571 + AL606834.1 *∗* 0.2287 + LINC00707 *∗* 0.2059 + OGFRP1 *∗* 0.2934 + U91328.1 *∗* −0.4533 + GASAL1 *∗* 0.3135.” The overview of the training cohort is shown in Figure 3(d). As shown in the KM survival curve, high-risk patients have a poorer prognosis than low-risk patients (Figure 3(e)). The satisfactory prediction performance of our model was shown by ROC curves (Figures 3(f)–3(h); 1-year AUC = 0.771, 3-year AUC = 0.741, 5-year AUC = 0.753). There were also more deaths in the high-risk group (Figure 3(i)). Compared to low-risk patients, high-risk patients had a worse survival rate (Figure 3(j)). ROC curves also indicated good prediction performance of our model in the validation group (Figures 3(k)–3(m); 1-year AUC = 0.678, 3-year AUC = 0.746, 5-year AUC = 0.766). The prognosis effect of these model lncRNAs is shown in Figures S1–S3. In univariate and multivariate analyses, the risk score was an independent predictor of patient survival (Figure S4).

### 3.3. Clinical Correlation Analysis and Nomogram

**Figure 4:**
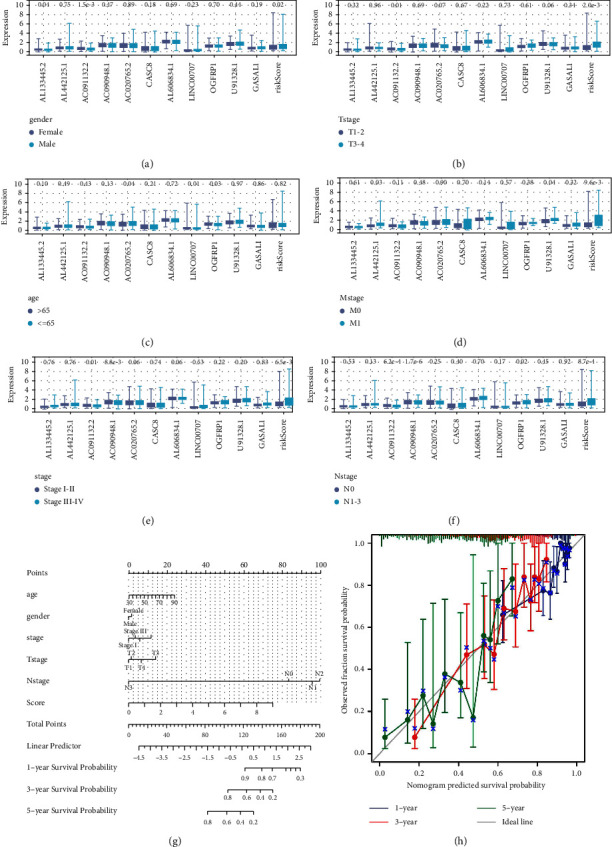
Clinical correlation analysis and nomogram. (a) Expression of model lncRNAs and risk score in patients with different gender; (b) expression of model lncRNAs and risk score in patients with different T stage; (c) expression of model lncRNAs and risk score in patients with different age groups; (d) expression of model lncRNAs and risk score in patients with different M stage; (e) expression of model lncRNAs and risk score in patients with different clinical stage; (f) expression of model lncRNAs and risk score in patients with different N stage; (g) a nomogram plot was constructed by combining clinical information and risk score; (h) calibration curves of 1-, 3- and 5-years.

Furthermore, clinical differences between high-risk and low-risk patients were explored (Figures 4(a)–4(f)). We found that AL133445.2 was upregulated, while AC091132.2 and risk score were downregulated in female patients (Figure 4(a)); the T3-4 patients have a higher risk score (Figure 4(b)); AC020765.2 was upregulated, while LINC00707 and OGFRP1 were downregulated in relatively young patients (Figure 4(c)); AL442125.1, U91328.1, and risk score were upregulated in M1 patients (Figure 4(d)); AC091132.2 and AC090948.1 were downregulated in stage III-IV patients (Figure 4(e)); AC091132.2 and AC090948.1 were downregulated, while OGFRP1 and risk score were upregulated in N1-3 patients (Figure 4(f)). The clinical information and risk score were combined to create a nomogram plot (Figure 4(g)). There was a satisfactory fit between the actual survival and the nomogram-predicted survival based on calibration curves (Figure 4(h)).

### 3.4. Biological Enrichment

**Figure 5:**
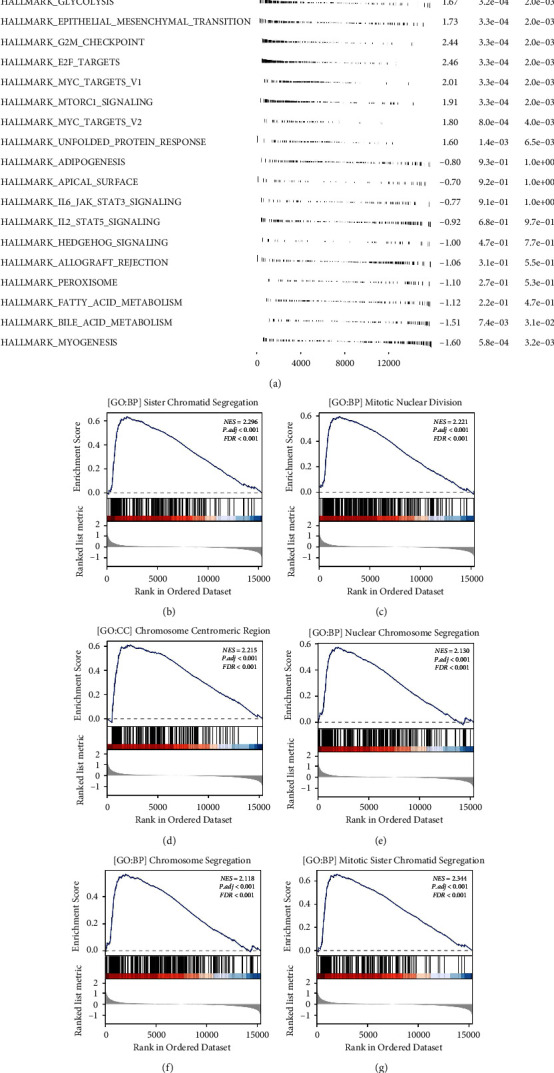
Biological enrichment analysis. (a) GSEA based on hallmark gene set; (b–g): GSEA based on GO gene set.

Next, biological differences between high- and low-risk groups were investigated. GSEA showed that the pathways of hypoxia, mitotic spindle, glycolysis, epithelial-mesenchymal transition (EMT), G2M checkpoint, MYC target, mTORC1 signaling and MYC target a were activated in high-risk patients (Figure 5(a)). For GO reference terms, the terms of sister chromatid segregation, mitotic nuclear division, chromosome centromeric region, nuclear chromosome segregation, chromosome segregation, and mitotic sister chromatid segregation were upregulated in the high-risk patients (Figures 5(b)–5(g)).

### 3.5. Immune-Related Analysis

**Figure 6:**
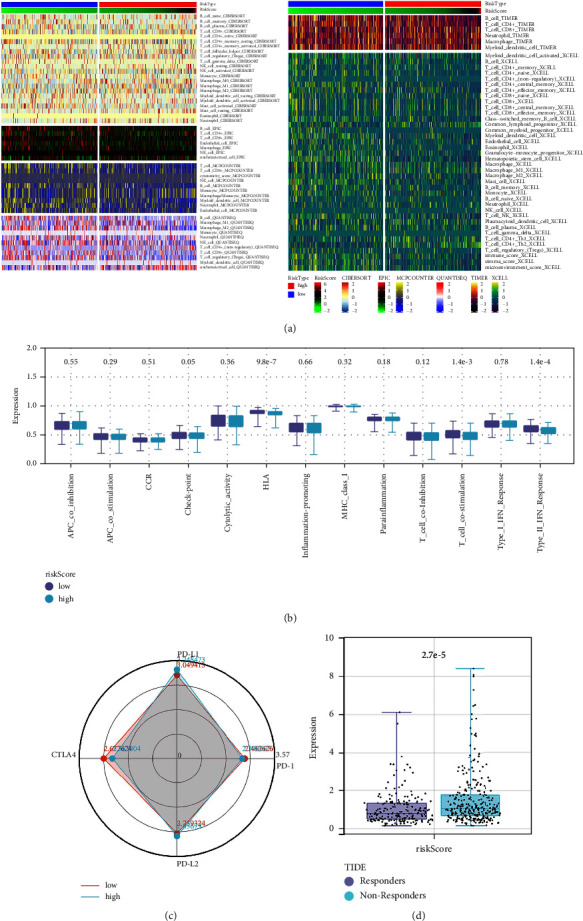
Immune-related analysis. (a) The tumor microenvironment of LUAD was quantified using multiple algorithms; (b) the quantified immune function by ssGSEA algorithm in high- and low-risk patients; (c) the key immune checkpoints in high- and low-risk patients; (d) the risk score in immunotherapy responders and nonresponders.

Then, we quantified the immune infiltration of LUAD tissue based on multiple algorithms, including XCELL, CIBERSORT, EPIC, MCPCOUNTER, QUANTISEQ, and TIMER. A positive correlation was found between risk score and monocyte, macrophage/monocyte, T_cell_CD4+_Th2, but a negative correlation with CD8+ T cells, CD4+ T cells, B cells, and NK cells (Figure 6(a)). Immune function analysis showed that in high-risk patients, the immune terms of type_II_IFN_response, check point, T_cell_costimulation, and HLA were downregulated, indicating that high-risk patients may have a lower immune function level (Figure 6(b)). For patients with LUAD, immunotherapy is an important treatment option. Therefore, we first explored the differences in key immune checkpoints (CTLA4, PD-1, PD-L1, PD-L2) in high- and low-risk patients. We noticed that CTLA4 has a higher expression level in low-risk patients (Figure 6(c)). Meanwhile, for other immune checkpoint genes, we noticed a higher expression level of CD276, TNFSF9, and HMGB1, while a lower level of BTN3A1, CD40LG, ENTPD1, HLA-DPA1, HLA-DPB1, HLA-DQA1, HLA-DQB2, HLA-DRA, ICAM1, ITGB2, SELP, SLAMF7, TIGIT, TNFRSF14, TNFRSF4, TNFSF15, TNFRSF25, CD48, and NRO1 in high-risk patients (Figure S5). According to the TIDE algorithm, immunotherapy responders may have a lower risk score (Figure 6(d)).

### 3.6. Genomic Instability and Drug Sensitivity Analysis

**Figure 7:**
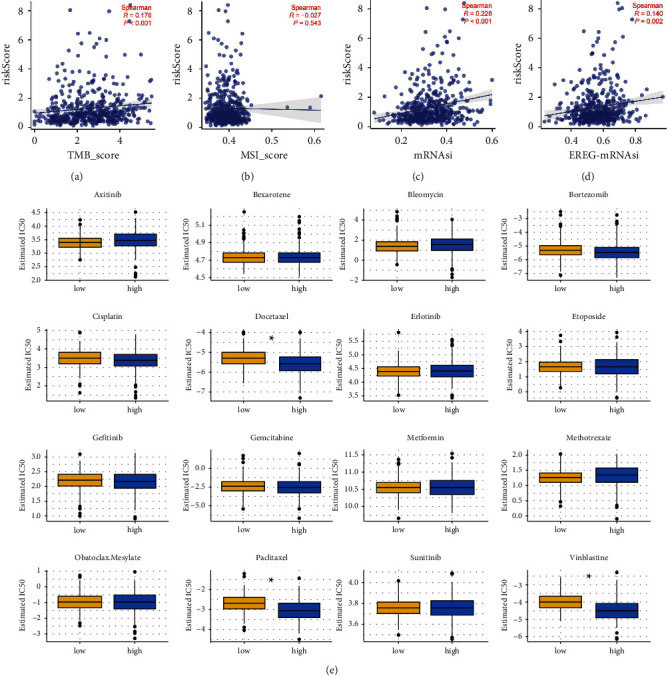
Genomic instability and drug sensitivity analysis. (a) Correlation between the risk score and TMB score; (b) correlation between the risk score and MSI score; (c) correlation between the risk score and mRNAsi; (d) correlation between the risk score and EREG-mRNAsi; (e) IC50 of specific drugs in high- and low-risk patients.

Genomic instability is another important factor affecting tumor progression. Therefore, we explored the genomic features in high- and low-risk patients. Results showed that risk score was positively correlated with TMB, mRNAsi, and EREG-mRNAsi, indicating that the patients with high-risk scores might have a worse genomic instability (Figures 7(a)–7(d)). In a drug sensitivity analysis, vinblastine, docetaxel, and paclitaxel seemed to be more sensitive to patients with high-risk cancers (Figure 7(e)).

### 3.7. Further Exploration of OGFRP1

**Figure 8:**
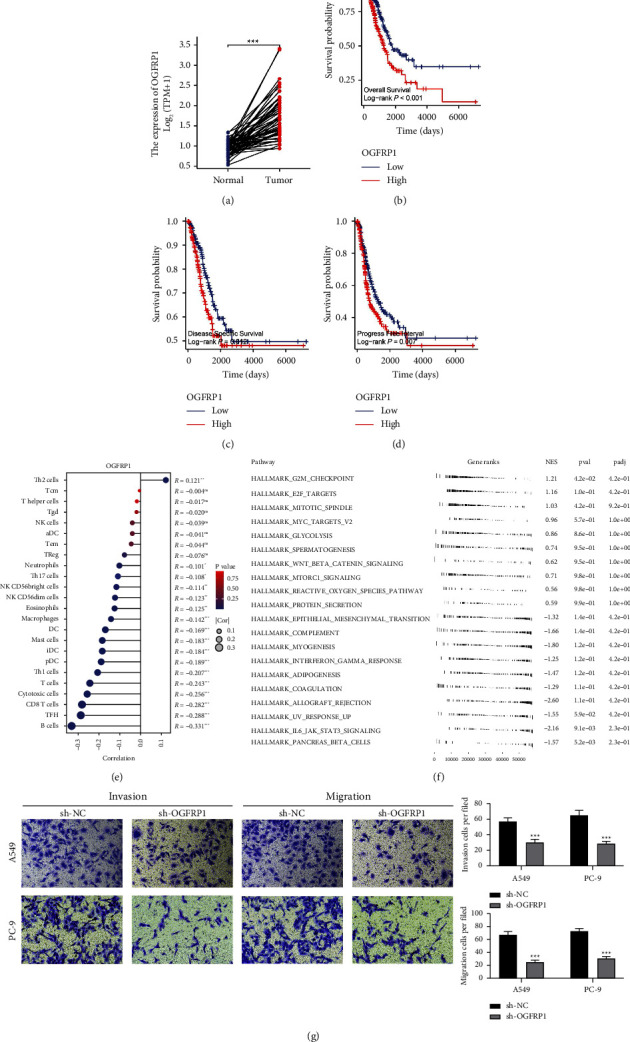
Role of OGFRP1 in LUAD. (a) Expression level of OGFRP1 in paired LUAD and normal tissue; (b–d): prognosis effect of OGFRP1 in LUAD; (e) immune infiltration analysis of OGFRP1; (f) GSEA of OGFRP1; (g) transwell assay of OGFRP1.

Then, we selected OGFRP1 for further analysis. We found the OGFRP1 was upregulated in LUAD tumor tissue (Figure 8(a)). KM survival curves showed that OGFR1 was associated with worse overall survival (OS), disease-free survival (DSS), and progression-free survival (PFI) (Figures 8(b)–8(d)). Results of ssGSEA showed that OGFRP1 was positively correlated with Th2 cells but negatively correlated with B cells, TFH, CD8+ T cells, cytotoxic cells, T cells, and Th1 cells (Figure 8(e)). Biological enrichment analysis showed that OGFRP1 was positively correlated with MYC targets, the mitotic spindle, E2F targets, G2M checkpoint, and glycolysis (Figure 8(f)). Clinical analysis showed a negative correlation between OGFRP1 and N stage. The knockdown efficiency of OGFRP1 is shown in Figure S6, and the sh#2 was selected for further experiments. Then, we performed a transwell assay. A significant reduction in lung cancer invasion and migration was observed when OGFRP1 was inhibited (Figure 8(g)).

## 4. Discussion

Globally, lung cancer remains a major public health concern. Lung cancer is a multifactorial disease whose pathogenesis remains unclear. With the development of molecular biology, people have gradually explored the mechanisms of cancer occurrence and development and developed promising targeted therapies for specific targets. Consequently, exploring possible targets at the molecular level is of great significance.

To the best of our knowledge, this is the first study to examine the role of disulfidptosis-related lncRNAs in LUAD. In our study, we identified 1401 lncRNAs significantly correlated with disulfidptosis-related genes (|Cor| > 0.3 and *P*  <  0.05). Then, we constructed a prognosis model consisting of 11 disulfidptosis-related lncRNAs, including AL133445.2, AL442125.1, AC091132.2, AC090948.1, AC020765.2, CASC8, AL606834.1, LINC00707, OGFRP1, U91328.1, and GASAL1. Also, the risk score and clinical information were combined to develop a nomogram. Analyses of biological enrichment and immune-related data were used to identify underlying differences between patients at high-risk and low-risk. Moreover, we noticed that the immunotherapy nonresponders have higher risk scores. Meanwhile, patients at high risk responded more strongly to docetaxel, paclitaxel, and vinblastine. Furthermore, further analysis of the model lncRNA OGFRP1 was conducted, including clinical, immune infiltration, biological enrichment analysis, and transwell assay. We discovered that by inhibiting OGFRP1, the invasion and migration abilities of lung cancer cells could be remarkably hindered.

Our results identified the role of 11 model lncRNAs in LUAD, which are associated with the disulfidptosis process. LncRNAs have been implicated in cancer in some cases. For example, the lncRNA AC090948.1 was found to be related to lipid metabolism, cuproptosis, and immunity in cancers [25–27]. Hu et al. noticed that AC020765.2 is related to autophagy in lung cancer [28]. Jiang et al. discovered that the inhibition of CASC8 could affect lung cancer progression and osimertinib sensitivity in a FOXM1-dependent manner [29]. Moreover, Zheng et al. found that AL606834.1 was associated with ferroptosis in lung cancer [30]. Ma et al. demonstrated that LINC00707 can promote lung cancer development by regulating Cdc42 [31]. Our results indicated that these model lncRNA are associated with the disulfidptosis process, which might provide a novel understanding of their role in lung cancer.

GSEA showed that the pathways of hypoxia, mitotic spindle, glycolysis, EMT, G2M checkpoint, E2F target, MYC target, mTORC1 signaling, and MYC target were activated in high-risk patients. Local hypoxia is an important characteristic of tumors. In lung cancer, Shi et al. found that YTHDF1 is associated with hypoxia adaptation, as well as lung cancer progression [32]. Zhang et al. noticed that in the absence of oxygen, bone marrow-derived mesenchymal stem cells can induce lung cancer metastasis through exosomal miRNAs and EMT pathways [33]. Yang et al. discovered that the FOXP3 could activate the Wnt/*β*-catenin signaling and EMT to promote lung cancer malignant phenotypes [34]. Liu et al. noticed that EMT can be activated by IL-6 depending on the NF-*κ*B/TIM-4 axis, therefore, facilitating lung cancer metastasis [35]. Liu et al. found that the interaction between TRIB2 and PKM2 can promote lung cancer progression by regulating the aerobic glycolysis process [36]. Hua et al. demonstrated that lncRNA-AC020978 induced by hypoxia can enhance lung cancer development through glycolytic metabolism regulated by the PKM2/HIF-1*α* axis [37]. Tantai et al. discovered that PHLPP2 ubiquitylation can be modified by TRIM46, therefore, enhancing lung cancer glycolysis and chemoresistance [38].

The influence of risk score on immune infiltrating cells may be one of the reasons for the prognosis differences in different risk groups. Zhang et al. noticed that the macrophage polarization regulated by SPP1 can lead to immune escape in LUAD [39]. Chen et al. discovered that exosomal-circUSP7 derived from lung cancer cells can result in CD8+ T cell dysfunction, therefore, affecting the efficiency of anti-PD-L1 therapy [40]. Fang et al. found that IDO1 could downregulate NKG2D to hamper NK cells function, further inhibiting lung cancer development [41].

Although our analysis is based on high-quality data and rigorous analysis, some limitations cannot be ignored. First, the list of disulfidptosis-related genes was collected from the previous study conducted by Liu and their colleagues. However, with the deepening of relevant research, there will be more and more potential genes that regulate defective protein synthesis. Second, immune infiltration analysis is performed using a variety of bioinformatics algorithms. However, bioinformatics algorithms cannot fully quantify the actual situation inside tumors.

## Data Availability

The datasets generated and/or analyzed during the current study are available in The Cancer Genome Atlas database repository (https://portal.gdc.cancer.gov/) and are available from the corresponding author on reasonable request.

## Supplementary Materials

Supplementary Materials Figure S1. KM survival curves of model lncRNAs (OS) in LUAD Notes: (A) KM survival curves of GYS1 in LUAD (OS); (B) KM survival curves of LRPPRC in LUAD (OS); (C) KM survival curves of NCKAP1 in LUAD (OS); (D) KM survival curves of NDUFA11 in LUAD (OS); (E) KM survival curves of NDUFS1 in LUAD (OS); (F) KM survival curves of NUBPL in LUAD (OS); (G) KM survival curves of OXSM in LUAD (OS); (H) KM survival curves of RPN1 in LUAD (OS); (I) KM survival curves of SLC3A2 in LUAD (OS); (J) KM survival curves of SLC7A11 in LUAD (OS). Figure S2. KM survival curves of model lncRNAs (DSS) Notes: (A) KM survival curves of GYS1 in LUAD (DSS); (B) KM survival curves of LRPPRC in LUAD (DSS); (C) KM survival curves of NCKAP1 in LUAD (DSS); (D) KM survival curves of NDUFA11 in LUAD (DSS); (E) KM survival curves of NDUFS1 in LUAD (DSS); (F) KM survival curves of NUBPL in LUAD (DSS); (G) KM survival curves of OXSM in LUAD (DSS); (H) KM survival curves of RPN1 in LUAD (DSS); (I) KM survival curves of SLC3A2 in LUAD (DSS); (J) KM survival curves of SLC7A11 in LUAD (DSS). Figure S3. KM survival curves of model lncRNAs (PFI) Notes: (A) KM survival curves of GYS1 in LUAD (PFI); (B) KM survival curves of LRPPRC in LUAD (PFI); (C) KM survival curves of NCKAP1 in LUAD (PFI); (D) KM survival curves of NDUFA11 in LUAD (PFI); (E) KM survival curves of NDUFS1 in LUAD (PFI); (F) KM survival curves of NUBPL in LUAD (PFI); (G) KM survival curves of OXSM in LUAD (PFI); (H) KM survival curves of RPN1 in LUAD (PFI); (I) KM survival curves of SLC3A2 in LUAD (PFI); (J) KM survival curves of SLC7A11 in LUAD (PFI). Figure S4. Univariate and multivariate analysis of risk score Notes: (A) Univariate analysis of risk score; (B) Multivariate analysis of risk score. Figure S5. The expression level of immune checkpoint molecules in high- and low-risk patients. Figure S6. Knockdown efficiency of OGFRP1 in A549 and PC-9 cell lines Notes: (A) Knockdown efficiency of OGFRP1 in A549 cell line; (B) Knockdown efficiency of OGFRP1 in PC-9 cell line.
