# Supplementary material for: Characterization and Prognosis of Biological Microenvironment in Lung Adenocarcinoma through a Disulfidptosis-Related lncRNAs Signature
Source: Genet Res (Camb). 2023 Aug 4;2023:6670514. doi: 10.1155/2023/6670514 (PMC10421709; doi:10.1155/2023/6670514)
Supplement: Supplementary Materials — Figure S1. KM survival curves of model lncRNAs (OS) in LUAD Notes: (A) KM survival curves of GYS1 in LUAD (OS); (B) KM survival curves of LRPPRC in LUAD (OS); (C) KM survival curves of NCKAP1 in LUAD (OS); (D) KM survival curves of NDUFA11 in LUAD (OS); (E) KM survival curves of NDUFS1 in LUAD (OS); (F) KM survival curves of NUBPL in LUAD (OS); (G) KM survival curves of OXSM in LUAD (OS); (H) KM survival curves of RPN1 in LUAD (OS); (I) KM survival curves of SLC3A2 in LUAD (OS); (J) KM survival curves of SLC7A11 in LUAD (OS). Figure S2. KM survival curves of model lncRNAs (DSS) Notes: (A) KM survival curves of GYS1 in LUAD (DSS); (B) KM survival curves of LRPPRC in LUAD (DSS); (C) KM survival curves of NCKAP1 in LUAD (DSS); (D) KM survival curves of NDUFA11 in LUAD (DSS); (E) KM survival curves of NDUFS1 in LUAD (DSS); (F) KM survival curves of NUBPL in LUAD (DSS); (G) KM survival curves of OXSM in LUAD (DSS); (H) KM survival curves of RPN1 in LUAD (DSS); (I) KM survival curves of SLC3A2 in LUAD (DSS); (J) KM survival curves of SLC7A11 in LUAD (DSS). Figure S3. KM survival curves of model lncRNAs (PFI) Notes: (A) KM survival curves of GYS1 in LUAD (PFI); (B) KM survival curves of LRPPRC in LUAD (PFI); (C) KM survival curves of NCKAP1 in LUAD (PFI); (D) KM survival curves of NDUFA11 in LUAD (PFI); (E) KM survival curves of NDUFS1 in LUAD (PFI); (F) KM survival curves of NUBPL in LUAD (PFI); (G) KM survival curves of OXSM in LUAD (PFI); (H) KM survival curves of RPN1 in LUAD (PFI); (I) KM survival curves of SLC3A2 in LUAD (PFI); (J) KM survival curves of SLC7A11 in LUAD (PFI). Figure S4. Univariate and multivariate analysis of risk score Notes: (A) Univariate analysis of risk score; (B) Multivariate analysis of risk score. Figure S5. The expression level of immune checkpoint molecules in high- and low-risk patients. Figure S6. Knockdown efficiency of OGFRP1 in A549 and PC-9 cell lines Notes: (A) Knockdown efficiency of OGFRP1 in A549 cell line; (B) Knockdown efficienc [file 6670514.f1.zip › Figure S4.pdf]

Figure S4

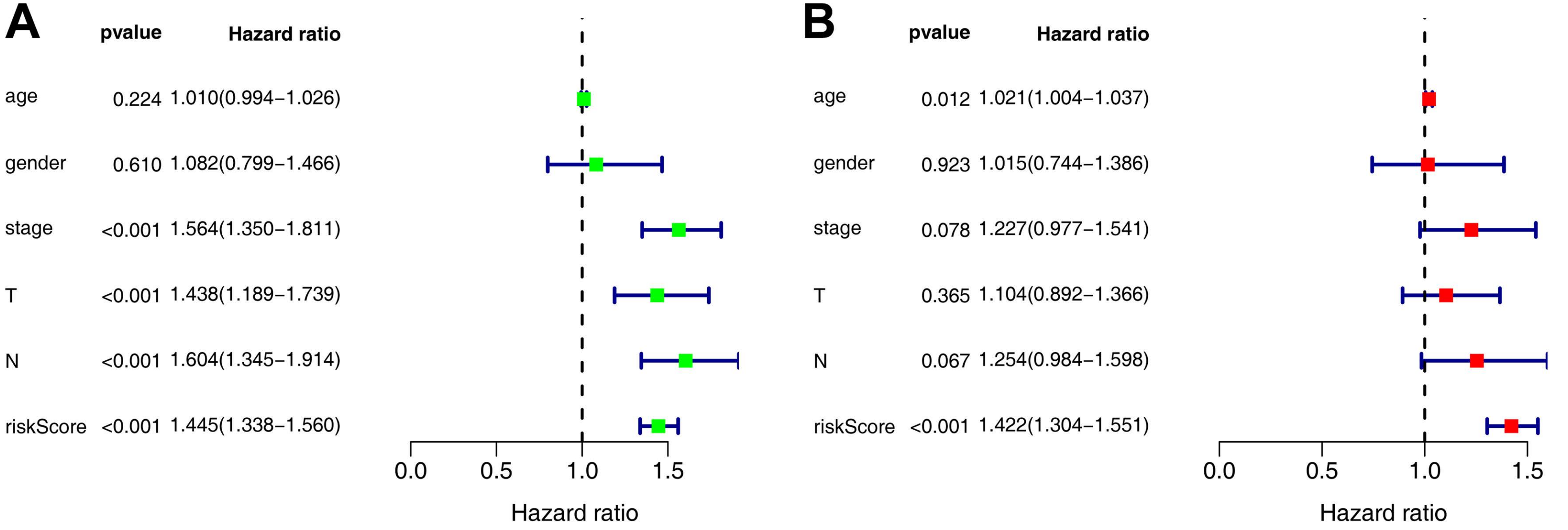
A
pvalue
Hazard ratio
age 0.224 1.010(0.994−1.026)
gender 0.610 1.082(0.799−1.466)
stage <0.001 1.564(1.350−1.811)
T <0.001 1.438(1.189−1.739)
N <0.001 1.604(1.345−1.914)
riskScore <0.001 1.445(1.338−1.560)
0.0
0.5
1.0
1.5
Hazard ratio
B
pvalue
Hazard ratio
age 0.012 1.021(1.004−1.037)
gender 0.923 1.015(0.744−1.386)
stage 0.078 1.227(0.977−1.541)
T 0.365 1.104(0.892−1.366)
N 0.067 1.254(0.984−1.598)
riskScore <0.001 1.422(1.304−1.551)
0.0
0.5
1.0
1.5
Hazard ratio
